# Comprehensive analysis of m6A RNA methylation regulators and the immune microenvironment in spinal cord injury

**DOI:** 10.3389/fneur.2026.1759661

**Published:** 2026-05-20

**Authors:** Xiaoqin Liu, Jiating Hu, Guodong Shi, Wenxia Zhu, Qiao Hao

**Affiliations:** 1 Yan’an Medical College of Yan’an University, Yan’an, China; 2 Department of Radiology, The Affiliated Hospital of Yan’an University, Yan’an, China

**Keywords:** bioinformatics, m6A regulators, machine learning, single-cell sequencing, spinal cord injury

## Abstract

**Background:**

Spinal cord injury (SCI) is devastating neurological disorder that leads to severe physical disabilities, reduced quality of life, and a substantial socioeconomic burden. N6-methyladenosine (m6A) RNA modification has emerged as an important regulator of RNA metabolism and immune responses; however, its role in SCI remains poorly understood.

**Methods:**

Transcriptomic datasets were obtained from the Gene Expression Omnibus (GEO) to identify differentially expressed m6A regulators in SCI. Hub genes were screened using multiple machine learning algorithms and further validated in an independent dataset. Immune cell infiltration was assessed using single-sample gene set enrichment analysis (ssGSEA), and miRNA–gene–TF interaction networks were constructed using NetworkAnalyst. Single-cell RNA sequencing (scRNA-seq) data were analyzed to characterize the cellular distribution of candidate genes. Finally, the expression of candidate genes was validated in a rat SCI model using quantitative real-time PCR (qRT-PCR) and immunofluorescence staining.

**Results:**

Fourteen differentially expressed m6A regulators were identified, among which eight candidate genes were selected using machine learning approaches. FTO and YTHDC1 were further identified as hub genes through validation in an independent dataset. Immune infiltration analysis revealed significant alterations in immune cell composition in SCI, and both FTO and YTHDC1 were significantly associated with multiple immune cell subsets. Consistent with increased m6A activity in microglia and astrocytes observed in scRNA-seq analysis, *Fto* and *Ythdc1* were highly expressed in these cell types as well as in granulocytes. Furthermore, *in vivo* experiments validated the downregulation of FTO and YTHDC1 in injured spinal cord tissue.

**Conclusion:**

These findings suggest that FTO and YTHDC1 may play important roles in the pathogenesis of SCI and represent potential biomarkers and therapeutic targets for further investigation.

## 1 Introduction

Spinal cord injury (SCI) remains a major challenge in contemporary medicine due to its profound impact on physical function, quality of life, and the substantial socioeconomic burden it imposes on healthcare systems (1, 2). The consequences of SCI are often severe, frequently resulting in permanent disabilities that require extensive medical care and long-term rehabilitation (3, 4). Current treatment strategies primarily include surgical decompression, rehabilitation, and neuroprotective agents (5). However, these approaches often result in limited functional recovery, underscoring the urgent need for novel therapeutic strategies targeting the underlying molecular mechanisms of SCI.

N6-methyladenosine (m6A) is a methyl modification at the N6 position of adenosine in RNA (6). As the most abundant internal modification in eukaryotic mRNA, m6A plays a critical role in regulating RNA metabolism, including splicing, translation, and stability (7, 8). The dynamic regulation of m6A is mediated by three classes of proteins— “writers,” “erasers,” and “readers,” which catalyze its deposition, removal, and recognition, respectively (9). Increasing evidence indicates that m6A modification is involved in a wide range of biological processes, including immune regulation and neurological disease pathogenesis (10–12). Notably, dysregulation of m6A regulators has been implicated in several neurodegenerative diseases (13). Emerging evidence suggests that m6A modification plays a critical role in SCI. Mechanistically, METTL3-mediated m6A modification regulates SCI progression by modulating miRNA-dependent autophagy pathways, such as the miR-30c/ATG5 axis (14). In addition, transcriptome-wide analyses have revealed dynamic changes in m6A methylation patterns in the spinal cord following nerve injury, implicating its involvement in inflammation, apoptosis, and neurogenesis (15). Furthermore, m6A regulatory proteins have also been shown to modulate neuronal apoptosis in SCI; for instance, VIRMA promotes neuronal apoptosis by inducing m6A methylation of STK10 (16).

Despite these advances, the specific role of m6A RNA methylation to SCI remains largely unclear. Elucidating the contribution of m6A regulators to SCI pathophysiology may provide novel insights into disease mechanisms and identify potential therapeutic targets for improving neurological recovery.

**Figure 1:**
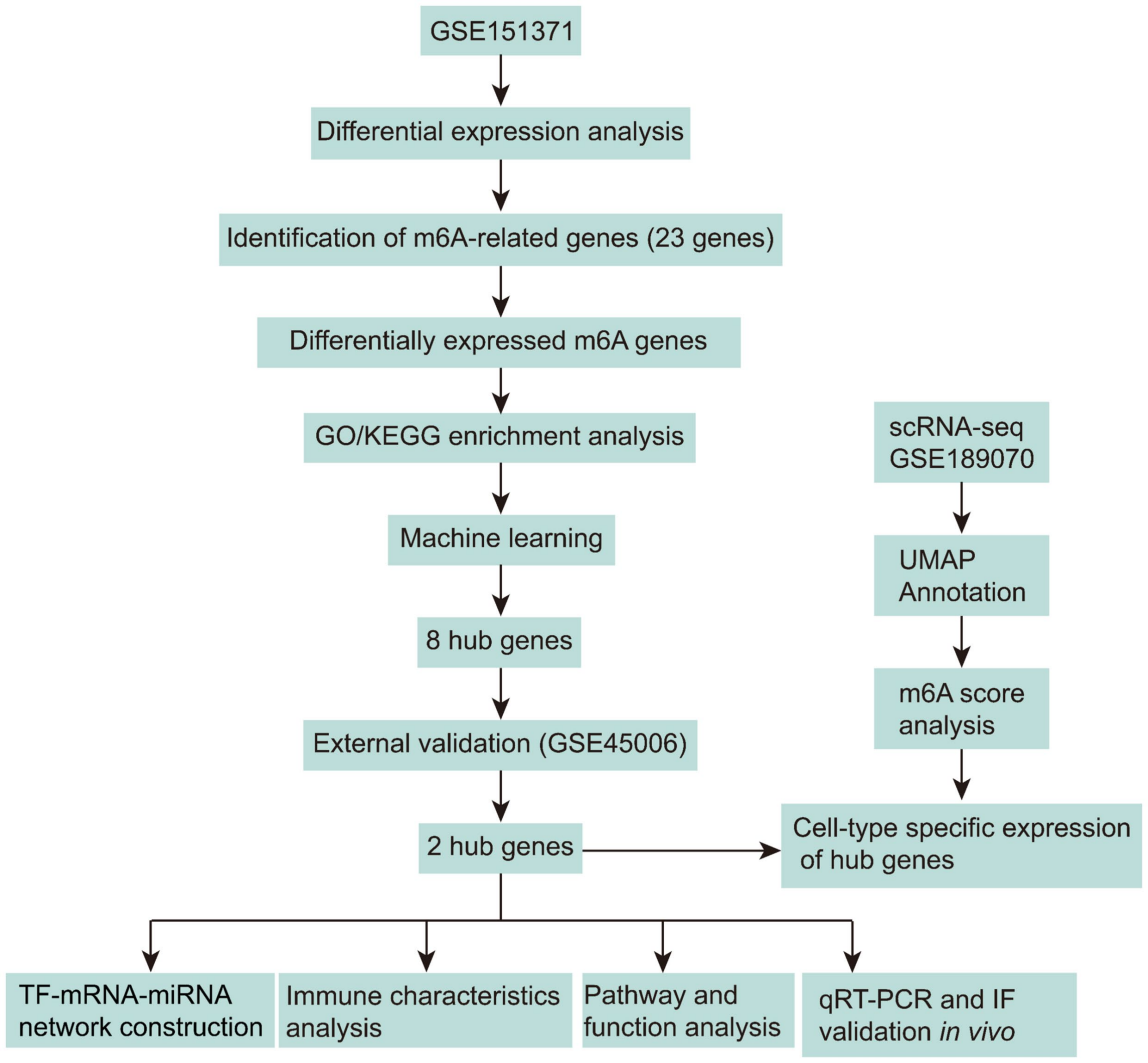
Schematic overview of the study design integrating bioinformatics analyses and experimental validation. Differentially expressed m^6^A-related genes were identified from GSE151371 and analyzed by GO/KEGG enrichment and machine learning to screen hub genes, followed by validation in GSE45006 and experimental confirmation. In parallel, scRNA-seq data (GSE189070) were used for cell annotation and m^6^A score analysis. Subsequent analyses included immune characteristics, regulatory network construction, and functional pathway analysis. Key genes were subsequently confirmed by RT-PCR and immunofluorescence (IF) *in vivo*.

In this study, we employed an integrative approach combining bioinformatics analyses, machine learning algorithms, and experimental validation to investigate the role of m6A regulators in SCI. By analyzing transcriptomic and single-cell RNA sequencing (scRNA-seq) data, we aimed to characterize the regulatory landscape and cellular distribution of m6A regulators in SCI. The integration of multi-omics data and cross-species validation enhances the robustness and translational relevance of our findings. Accordingly, we analyzed GEO datasets and validated the expression of identified hub genes in a rat SCI model to characterize the expression patterns of m6A regulators in SCI (Figure 1).

## 2 Materials and methods

### 2.1 Microarray datasets

SCI-related datasets (GSE151371, GSE189070, and GSE45006) were obtained from the Gene Expression Omnibus (GEO) database (https://www.ncbi.nlm.nih.gov/) (17). The GSE151371 dataset contains RNA-seq data from peripheral blood samples of 38 patients with SCI, 10 healthy controls (HC), and 10 non-CNS trauma controls. In the present study, only SCI patients and HC were included, while non-CNS trauma controls were excluded to avoid potential confounding effects related to systemic inflammatory responses induced by peripheral injury. According to the original TRACK-SCI study, blood samples were collected within a few hours after injury, representing the ultra-acute phase of SCI. However, the exact sampling time for each RNA-seq sample is not specified in the GEO metadata (18). Therefore, all SCI samples were analyzed collectively and defined as the early/acute SCI group, rather than being stratified by specific timepoints.

The GSE45006 dataset includes RNA microarray data from rat spinal cord tissues collected at multiple post-injury timepoints. In this study, samples obtained at 1, 3, and 7 days post-injury were selected, as these timepoints are commonly used to capture early pathological and inflammatory responses following SCI.

The GSE189070 dataset, generated using the GPL24247 platform, contains single-cell RNA sequencing data from mouse spinal cord tissues, including both uninjured and injured samples across multiple timepoints (19). Consistently, cells from samples collected at 1, 3, and 7 days post-injury were selected for downstream analysis to investigate the cellular expression patterns of candidate genes. These timepoints were chosen to ensure a consistent focus on the early phase of SCI across datasets and to facilitate cross-platform and cross-species comparisons.

### 2.2 Investigation of the m6A expression pattern

Based on previous research, 23 m6A-related genes were selected for analysis (20, 21), including six writers, two erasers, and fifteen readers. The detailed list of these regulators is provided in Table 1. Expression levels of m6A regulators between HC and SCI groups in the GSE151371 dataset were compared using the Wilcoxon rank-sum test implemented in R. Genes with *p*-value < 0.05 were considered statistically significant. The expression patterns of these differentially expressed regulators were visualized using heatmaps generated with the “pheatmap” package (version 1.0.12).

**Table 1:** List of m6A regulators included in this study.

Category	Gene symbols
Writers (6)	METTL3, METTL14, WTAP, ZC3H13, RBM15B, CBLL1
Erasers (2)	ALKBH5, FTO
Readers (15)	YTHDC1, YTHDC2, YTHDF1, YTHDF2, YTHDF3, HNRNPC, FMR1, LRPPRC, HNRNPA2B1, IGFBP1, IGFBP2, IGFBP3, RBMX, ELAVL1, IGF2BP1

### 2.3 Calculation of the m6A score based on PCA

To quantify the m6A modification pattern in each sample, principal component analysis (PCA) was performed based on the expression of differentially expressed m6A regulators to construct an m6A scoring system (22). The m6A score for each sample was calculated as follows: m6Ascore=∑(pc1i+pc2i) , where PC1 and PC2 represent the first and second principal components derived from PCA, and 𝑃𝐶1𝑖 and 𝑃𝐶2𝑖 denote the corresponding scores of gene i on the first and second principal components, respectively.

### 2.4 Functional enrichment analysis

Gene Ontology (GO) and Kyoto Encyclopedia of Genes and Genomes (KEGG) enrichment analyses were performed to explore the biological functions and pathways associated with differentially expressed genes (23, 24). Enrichment analyses were conducted using the “clusterProfiler” R package (25). The Benjamini–Hochberg (BH) method was applied for multiple testing correction. Terms with *p*-value < 0.05 and q-value (false discovery rate, FDR) < 0.1 were considered statistically significant.

### 2.5 Identification of key m6A regulators using machine learning algorithms

Fourteen differentially expressed m6A regulators were subjected to feature selection using eight machine learning algorithms, including Least Absolute Shrinkage and Selection Operator (LASSO) with 100 iterations and 10-fold cross-validation, Learning Vector Quantification (LVQ), Boruta, Bagged decision trees (Bagged Trees), Random Forest-based recursive feature elimination (RF-RFE), Naive Bayes-based recursive feature elimination (NB-RFE), Support Vector Machine with radial basis function kernel (SVM-RBF), and eXtreme Gradient Boosting (XGBoost). For LASSO analysis, logistic regression or Cox proportional hazards regression models were applied depending on the outcome type. The Boruta algorithm was employed to identify relevant features by comparing the importance of original attributes with that of shadow features. The overlap of candidate genes identified by different algorithms was visualized using UpSet plot implemented in the “UpSet” package (version 1.4.0). Hub genes were defined as those selected by at least four of the eight algorithms, representing a majority voting strategy to improve robustness and reduce method -specific bias.

### 2.6 Functional pathway analysis of the key m6A regulators

To investigate the biological functions and regulatory pathways associated with key m6A regulators, single-gene gene set enrichment analysis (GSEA) was performed. An adjusted *p*-value < 0.05 was considered statistically significant. The top five positively and negatively enriched pathways with the highest enrichment scores were visualized.

### 2.7 Immune cell infiltration analysis

The infiltration levels of 28 immune cell types in the GSE151371 dataset were estimated using the single-sample gene set enrichment analysis (ssGSEA) implemented in the “GSVA” R package (2.0.7), based on immune cell marker genes obtained from the CellMarker database. Pearson correlation analysis was performed within the SCI group to assess the associations between selected hub m6A regulators and differentially infiltrated immune cell types based on ssGSEA-derived infiltration scores, thereby minimizing potential confounding effects introduced by inter-group differences. In addition, correlation analysis among different immune cell types was performed within the SCI group to evaluate interactions within the immune microenvironment. To control for multiple comparisons, *p*-values were adjusted using the Benjamini–Hochberg false discovery rate (FDR) method.

### 2.8 miRNA-gene-TF interaction networks analysis

Interactions among hub m6A regulators, miRNAs, and transcription factors (TFs) were analyzed using the NetworkAnalyst 3.0 platform^1^ (26). The resulting miRNA-gene-TF interaction network was visualized using Cytoscape software (27).

### 2.9 Single-cell RNA-Seq analysis

Single-cell gene expression matrices were processed and analyzed using the “Seurat” R package (version 5.2.1). A total of 53,182 high-quality single cells were included after quality control and doublet removal, comprising 10,254 cells from uninjured samples and 42,928 cells from SCI samples across different time points (1, 3, and 7 days). Low-quality cells were filtered based on the number of detected genes, unique molecular identifier (UMI) counts, and the percentage of mitochondrial gene expression. The data were normalized using the “NormalizeData” function in “Seurat” and subjected to downstream analysis (28). Dimensionality reduction was performed using Uniform Manifold Approximation and Projection (UMAP), and cells were visualized in two-dimensional space. Cell clustering was conducted using the “FindNeighbors” and “FindClusters” functions in “Seurat,” and clusters were annotated based on canonical marker genes. To evaluate m6A regulatory activity at the single-cell level, the “AUCell_calcAUC” function from the “AUCell” package (version 1.28.0) was applied using a predefined gene set of m6A regulators, including writers, erasers, and readers. The resulting m6A score reflects the overall activity of the m6A regulatory machinery in each cell. Differences in m6A activity scores were further compared across different cell types.

### 2.10 Establishment of the SCI animal model

Twelve female Sprague–Dawley (SD) rats (220–240 g) were randomly divided into two groups: the Sham group and the SCI group. Outcome assessments were performed in a blinded manner. All animals were obtained from the Laboratory Animal Center of Xi’an Jiaotong University (Animal license No. SCXC [Shan] 2023–002). All experimental procedures were approved by the Animal Ethics Committee of Yan’an University and conducted in accordance with institutional and national guidelines for the care and use of laboratory animals. The SCI model was established using Allen’s method. Briefly, rats were anesthetized via intraperitoneal injection of 3% pentobarbital sodium (60 mg/kg) and placed on a surgical platform. A laminectomy was performed at the T10 vertebral level to expose the dorsal surface of the spinal cord. In the SCI group, a contusion injury was induced using a force of 200 kdyn/cm^2^ applied from a height of 3 cm. Rats in the Sham group underwent laminectomy without impact injury. Postoperatively, animals were housed individually and administered subcutaneous injections of penicillin (400,000 U/rat/day) for three consecutive days to prevent infection. Manual bladder expression was performed twice daily. Spinal cord tissue samples were collected 3 days after injury for subsequent analysis.

### 2.11 Quantitative real-time PCR (qRT-PCR)

Total RNA was extracted from rat spinal cord tissues using an RNA Extraction Kit (AC0201, SparkJade, China) according to the manufacturer’s instructions. Complementary DNA (cDNA) was synthesized using the SPARKscript II One Step RT-PCR Kit (AG0401, SparkJade, China).

Quantitative real-time PCR (qRT-PCR) was performed using a 2 × SYBR Green qPCR Mix (AH0102, SparkJade, China) to determine the expression levels of target genes. *β-actin* was used as an internal reference, and relative gene expression levels were calculated using the 2^–ΔΔCT^ method. The primer sequences used for amplification are listed in Table 2. All sample were analyzed in triplicate to ensure reproducibility.

**Table 2:** Sequences of primers used for qRT-PCR analysis.

Gene	Sequence (5′–3′)	Product size (bp)	Species
*Fto*	Forward: GGGAGCGGGAAGCTAAGAAA	135	Rat
	Reverse: CAGCCTCTCGGAAAACCAGT		
*Ythdc1*	Forward: ACTGACGGACGGACTCGT	146	Rat
	Reverse: TGGAGCAGTCTTGGCAATCT		
*β-actin*	Forward: CCCATCTATGAGGGTTACGC	150	Rat
	Reverse: TTTAATGTCACGCACGATTTC		

### 2.12 Immunofluorescence (IF)

Spinal cord tissues were post-fixed in 4% paraformaldehyde (PFA) overnight at 4 °C, followed by cryoprotected in 30% sucrose solution until complete dehydration. The tissues were then embedded in optimal cutting temperature (OCT) compound, and serial sections (10 μm) were prepared using a cryostat microtome (Leica, Germany). Sections were washed three times with phosphate-buffered saline (PBS) and blocked with 5% normal goat serum at room temperature for 1 h. Subsequently, sections were incubated overnight at 4 °C with the primary antibodies (YTHDC1, 1:200, Proteintech, 14,392-1-AP, China; FTO 1:200, Immunoway, PT0383R, USA). After washing with PBS, sections were incubated with appropriate fluorescent secondary antibodies (Abbkine, KTD109, China) at room temperature for 1 h in the dark. The sections were then washed again and counterstained with DAPI (Solarbio, C0065, China) for 10 min. Fluorescence images were acquired using a confocal laser scanning microscope. Quantification of mean fluorescence intensity (MFI) was performed using ImageJ software. Image analysis was performed in a blinded manner, with investigators unaware of the group allocation.

### 2.13 Statistics analysis

Statistical analyses were performed using R software (version 4.2.1) and GraphPad Prism (version 9.0). Pearson correlation analysis was used to assess associations between variables. Data are presented as the mean ± standard error of the mean (SEM). Differences between groups were analyzed using an unpaired Student’s *t*-test. A *p* < 0.05 was considered statistically significant.

## 3 Results

### 3.1 Expression patterns of m6A regulators in SCI and HC samples

**Figure 2:**
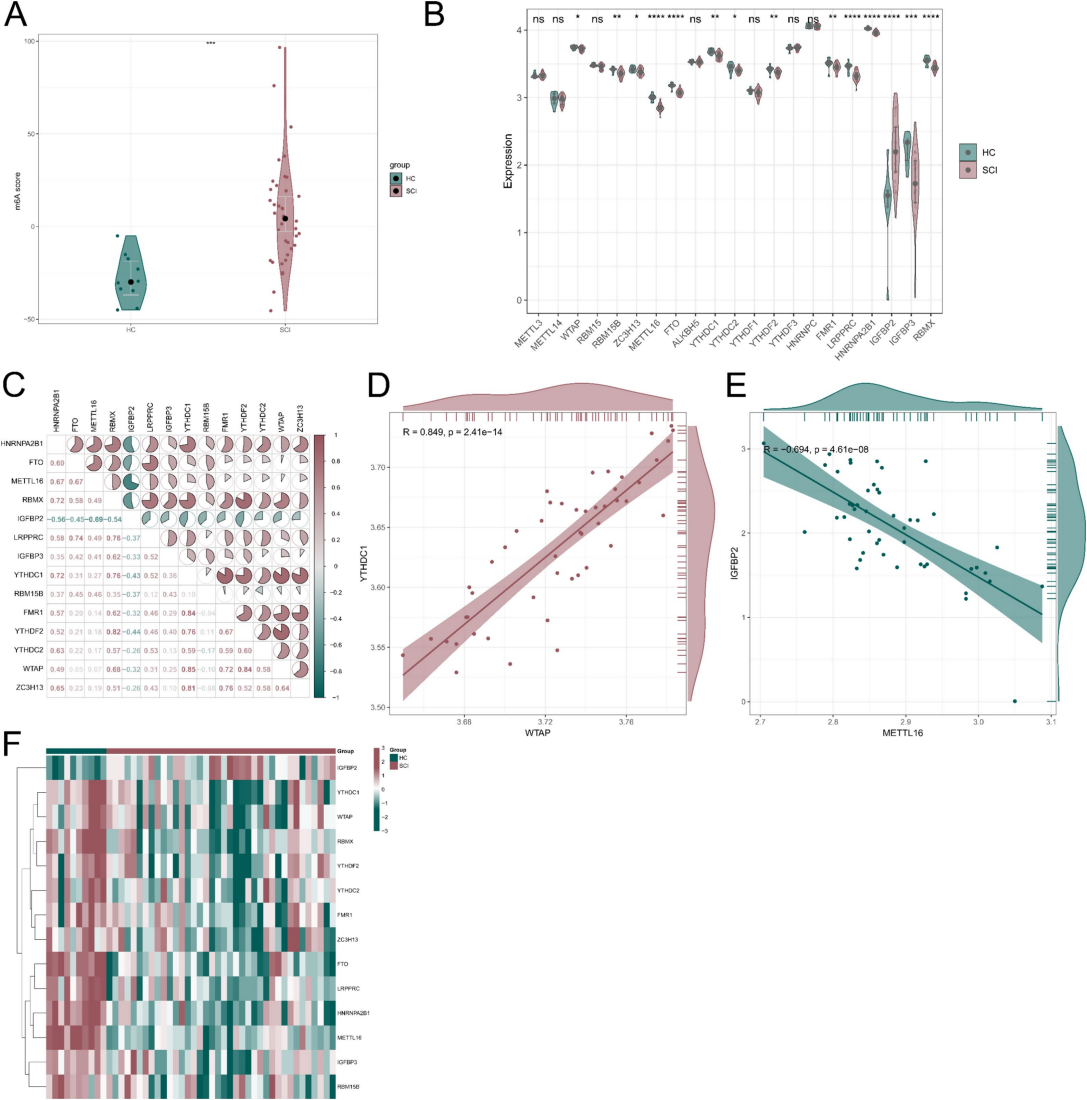
Expression profiles of m^6^A regulators in SCI and HC samples. **(A)** Comparison of m^6^A scores between SCI and HC samples. **(B)** Boxplots showing the expression of 23 m^6^A regulators in SCI and HC samples. **(C)** Pearson correlation analysis of 14 differentially expressed m^6^A regulators in SCI samples. (D, E) Scatter plots showing the most significant correlations among the 14 differentially expressed m^6^A regulators: including a positive correlation between WTAP and YTHDC1 and a negative correlation between METTL16 and IGFBP2. (F) Heatmap showing the expression patterns of the 14 differentially expressed m^6^A regulators in HC and SCI samples. *p < 0.05, **p < 0.01, and ***p < 0.001.

To investigate the expression patterns of 23 m6A regulators, differential expression analysis was performed between SCI and HC samples. PCA based on the expression of these regulators was used to calculate the m6A score for each sample. The m6A score was significantly higher in SCI samples compared with HC samples (Figure 2A). Among the 23 m6A regulators, IGFBP2 was significantly upregulated in the SCI group, whereas WTAP, RBM15B, ZC3H13, METTL16, FTO, YTHDC1, YTHDC2, YTHDF2, FMR1, LRPPRC, HNRNPA2B1, IGFBP3, and RBMX were significantly downregulated (Figure 2B). Correlation analysis of 14 differentially expressed m6A regulators revealed strong interrelationships among these regulators (Figure 2C). Notably, WTAP and YTHDC1 exhibited the strongest positive correlation (Pearson correlation coefficient = 0.849, Figure 2D), whereas METTL16 showed a negative correlation with IGFBP2 (Pearson correlation coefficient = −0.694, Figure 2E). A heatmap illustrating the expression patterns of the 14 differentially expressed m6A regulators in SCI and HC samples is shown in Figure 2F. These findings suggest that dysregulation of m6A regulators may contribute to the progression of SCI by altering RNA methylation patterns.

### 3.2 Functional enrichment analysis

**Figure 3:**
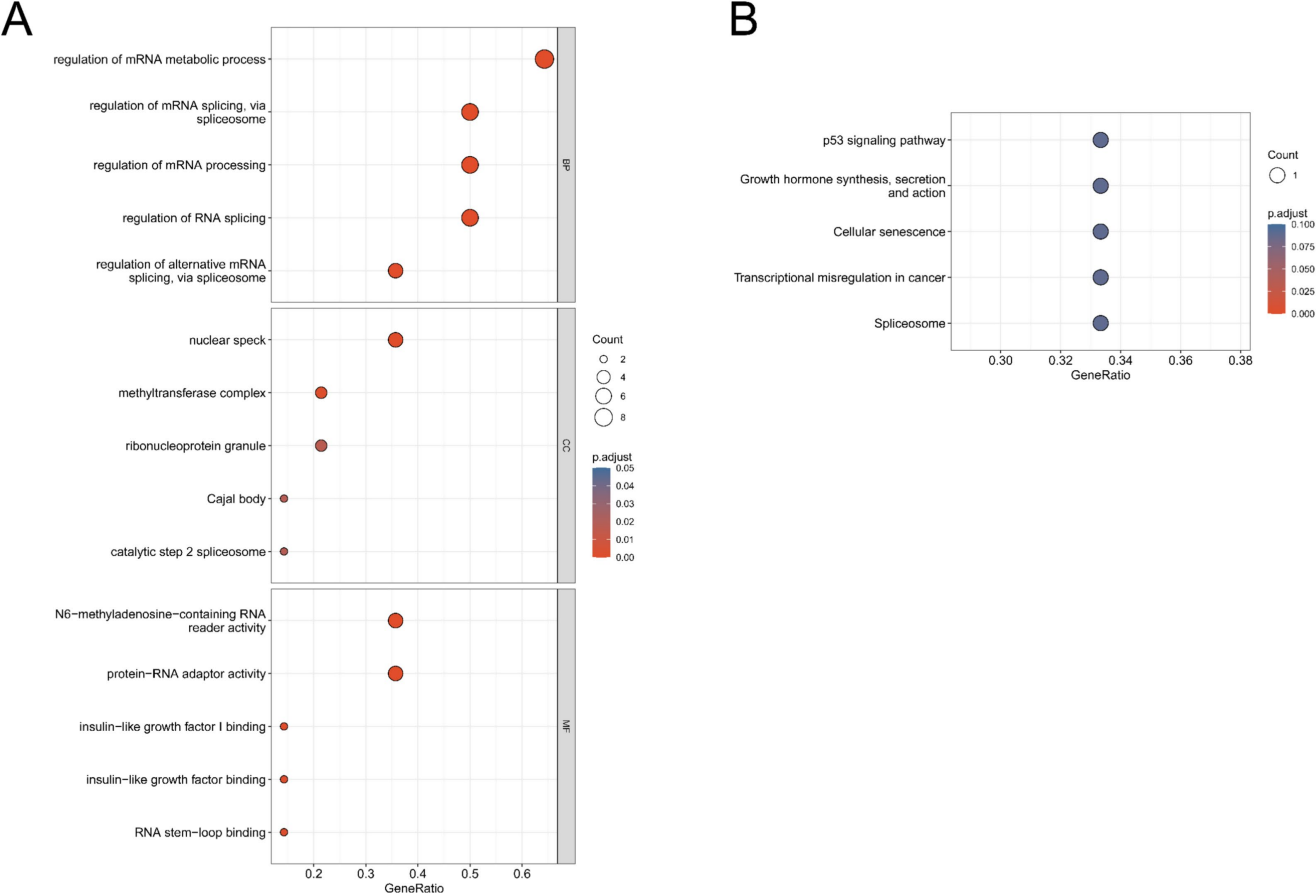
Functional enrichment analysis of the 14 differentially expressed m^6^A regulators. **(A)** GO enrichment analysis. **(B)** KEGG pathway analysis showing significantly enriched biological processes and pathway.

To explore the potential biological functions and pathways associated with m6A regulators, Gene Ontology (GO) and Kyoto Encyclopedia of Genes and Genomes (KEGG) enrichment analyses were performed based on the 14 differentially expressed m6A regulators. GO analysis revealed that these regulators are mainly involved in mRNA metabolic processes, mRNA splicing via the spliceosome, and RNA processing. In addition, they were enriched in functions such as N6-methyladenosine-containing RNA binding, protein-RNA adaptor activity, and cellular components including nuclear speck, methyltransferase complex, ribonucleoprotein granule, Cajal body, and catalytic step 2 spliceosome (Figure 3A). KEGG analysis further indicated that m6A regulators are associated with multiple biological pathways, including cellular senescence and RNA splicing, and are enriched in pathways such as the p53 signaling pathway and growth hormone signaling pathway (Figure 3B).

### 3.3 Identification of key m6A regulators using machine learning algorithms

**Figure 4:**
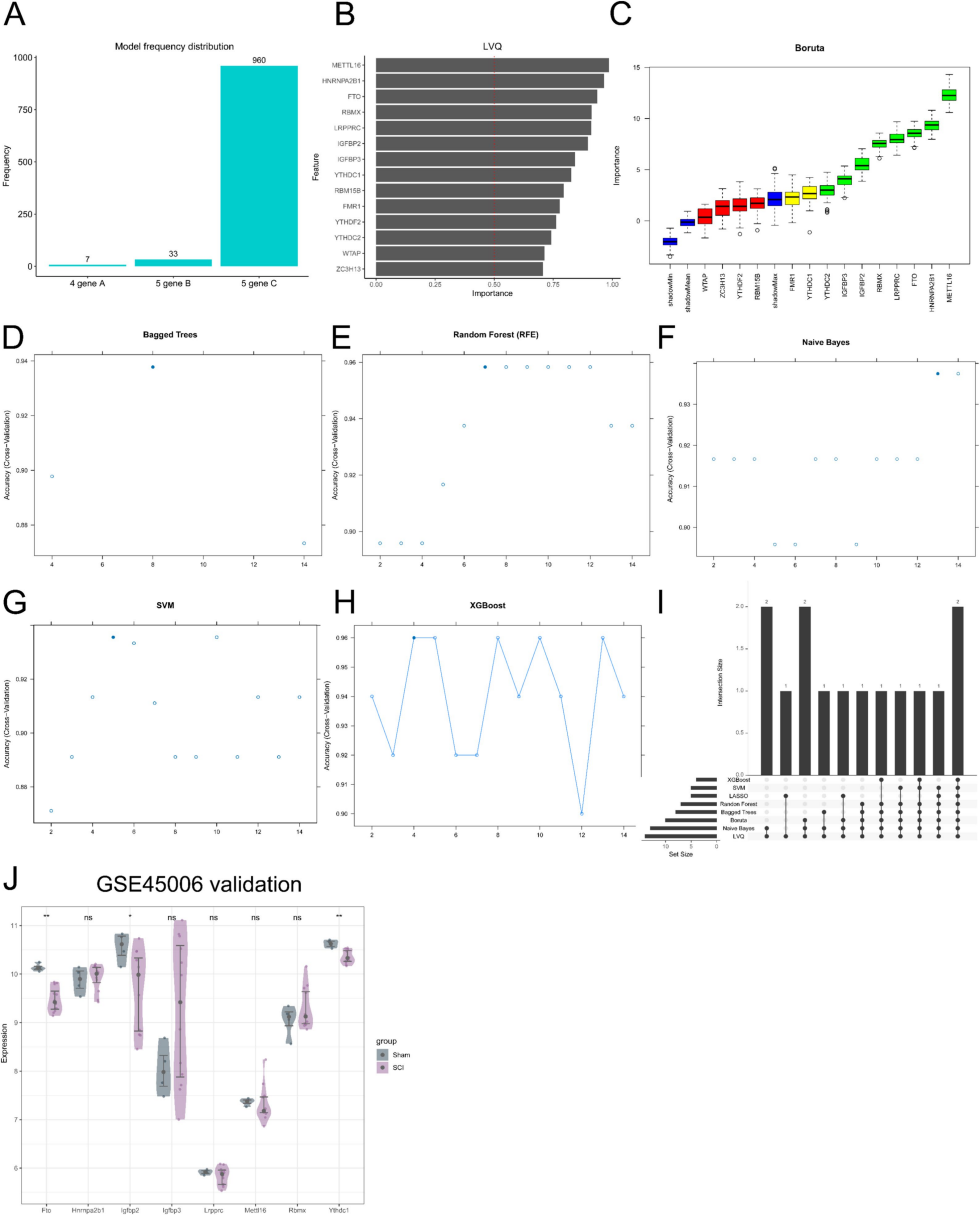
Identification of key m^6^A regulators using machine learning. **(A–H)** Results of eight machine learning algorithms based on the GSE151371 dataset: LASSO logistic regression **(A)**, LVQ **(B)**, Boruta **(C)**, Bagged Trees **(D)**, Random Forest-based recursive feature elimination (RF-RFE) **(E)**, Naive Bayes-based recursive feature elimination (NB-RFE) **(F)**, Support Vector Machine with radial basis function kernel (SVM-RBF) **(G)**, and XGBoost **(H)**. **(I)** UpSet plot showing the intersection of candidate genes identified by different algorithms; hub genes were defined as those identified by at least four algorithms. **(J)** Validation of hub genes in the GSE45006 dataset. *p < 0.05, **p < 0.01.

Eight machine learning algorithms were applied to identify key m6A regulators associated with SCI based on the GSE151371 dataset. Fourteen differentially expressed m6A regulators were subjected to feature selection using LASSO logistic regression with 100 iterations (Figure 4A), LVQ algorithm (Figure 4B), Boruta (Figure 4C), Bagged Trees (Figure 4D), RF-RFE (Figure 4E), NB-RFE (Figure 4F), SVM-RBF (Figure 4G), and XGBoost algorithm (Figure 4H). Using a majority voting strategy, genes identified by at least four of the eight algorithms were defined as candidate hub genes, resulting in eight genes (Figure 4I), including FTO, HNRNPA2B1, IGFBP2, IGFBP3, LRPPRC, METTL16, RBMX, and YTHDC1. To further validate these findings, the independent dataset GSE45006 was analyzed. The results showed that only *Fto*, *Igfbp2*, and *Ythdc1* were significantly differentially expressed between the SCI and Sham groups (Figure 4J). Notably, *Igfbp2* was downregulated in the SCI group in GSE45006 (Figure 4J), while it was upregulated in GSE151371 (Figures 2B,F), indicating dataset-dependent expression heterogeneity. Therefore, *Igfbp2* was excluded from subsequent analysis.

### 3.4 Immune infiltration analysis

**Figure 5:**
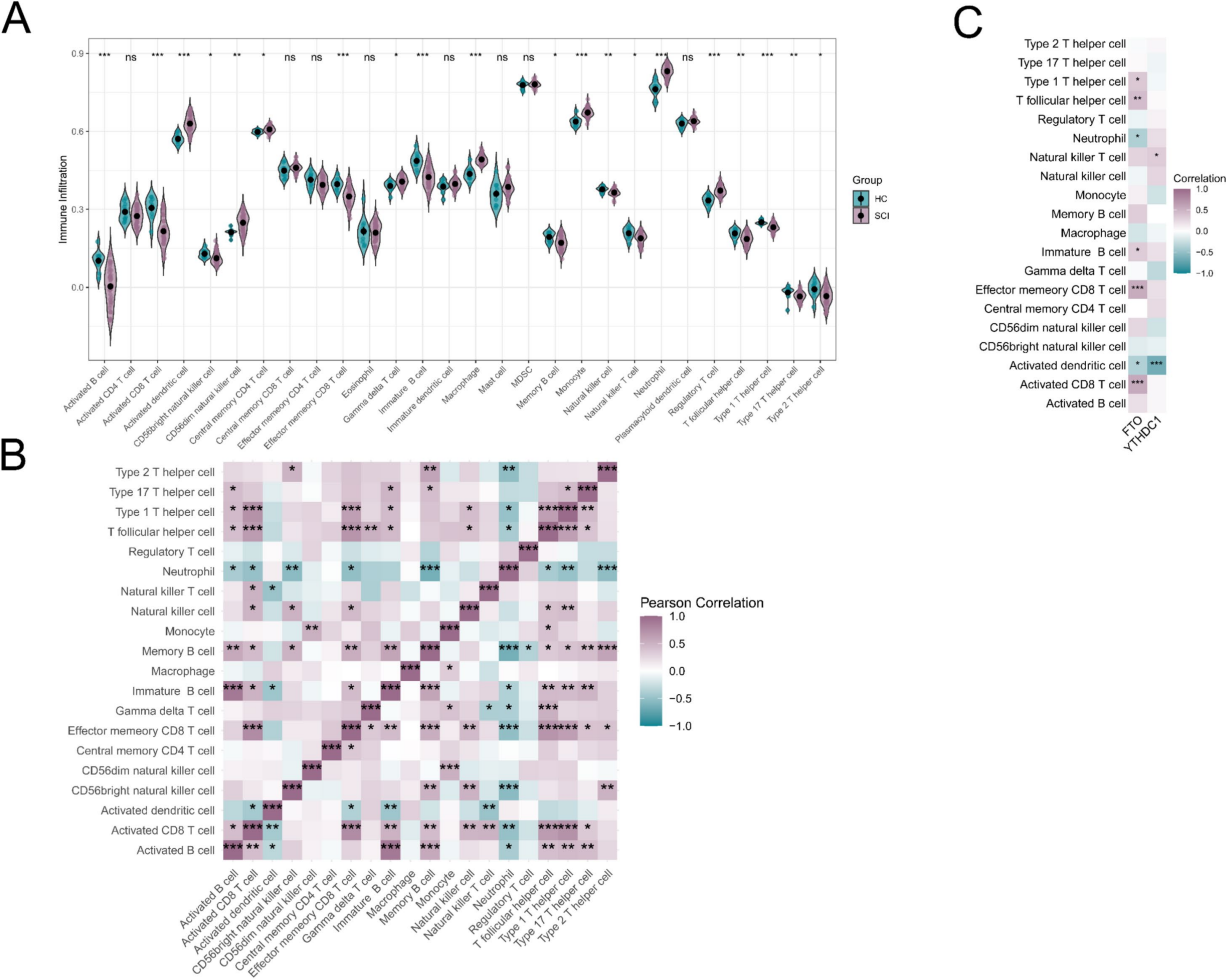
Immune microenvironment in SCI and HC groups. **(A)** Violin plot of the infiltration levels of 28 immune cell types in SCI and HC samples. **(B)** Pearson correlation analysis of immune cell interactions in the SCI group. **(C)** Pearson correlation analysis between key m^6^A regulators (FTO and YTHDC1) and immune cell infiltration levels in the SCI group. Statistical significance was determined using the Benjamini–Hochberg false discovery rate (FDR)-adjusted *p values (*p < 0.05, **p < 0.01, and ***p < 0.001).

The infiltration levels of 28 immune cell types were estimated using ssGSEA in both HC and SCI groups. Compared with the HC group, SCI samples showed significantly increased infiltration of eight immune cell types, including activated dendritic cells, CD56^dim natural killer cells, central memory CD4 T cells, gamma delta T cells, macrophages, monocytes, neutrophils, and regulatory T cells (Figure 5A). In contrast, 12 immune cell types, including activated B cells, activated CD8 T cells, CD56^bright natural killer cells, effector memory CD8 T cells, immature B cells, memory B cells, natural killer cells, natural killer T cells, T follicular helper cells, type 1 T helper cells, type 17 T helper cells, and type 2 T helper cells, were significantly enriched in the HC group (Figure 5A). Correlation analysis revealed predominantly positive correlations among immune cell types within the SCI group, while a subset of immune cells showed negative correlations with neutrophils and activated dendritic cells (Figure 5B). FTO expression was positively correlated with activated CD8 T cells, effector memory CD8 T cells, immature B cells, T follicular helper cells, and type 1 T helper cells, which were more abundant in the HC group (Figure 5C). In contrast, FTO expression was negatively correlated with activated dendritic cells and neutrophils, which were enriched in the SCI group (Figure 5C). Similarly, YTHDC1 expression was positively correlated with natural killer T cells and negatively correlated with activated dendritic cells (Figure 5C).

### 3.5 Pathway analysis of key m6A regulators

**Figure 6:**
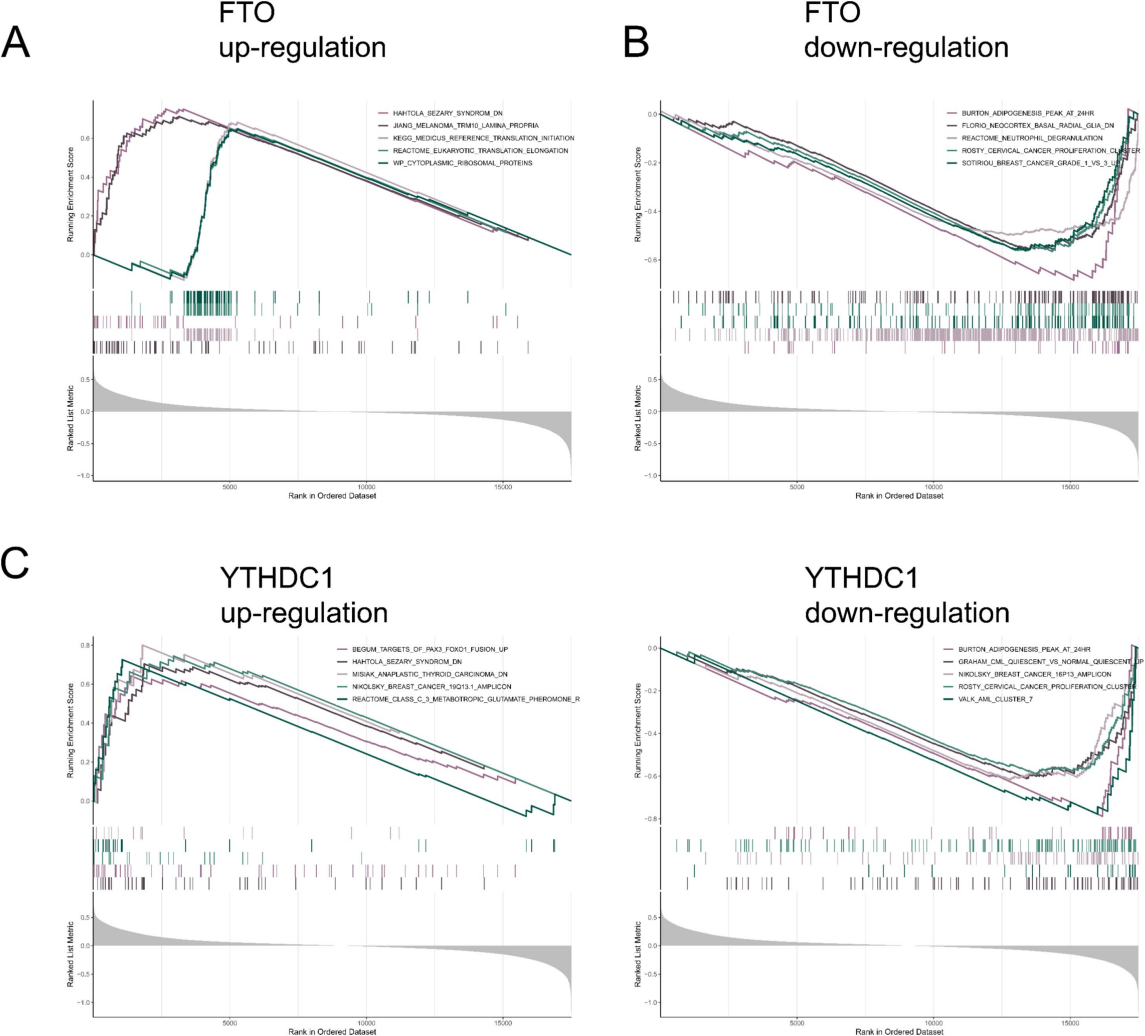
Pathway analysis of key m^6^A regulators. **(A,C)** Top five positively correlated pathways for FTO **(A)** and YTHDC1 **(C)**. **(B,D)** Top five negatively correlated pathways with FTO **(B)** and YTHDC1 **(D)**, based on gene expression correlation analysis.

A single-gene GSEA was performed to explore the biological functions and pathways associated with the key m6A regulators. GSEA results showed that FTO was positively associated with pathways related to translation initiation and elongation, as well as several cancer-related pathways, including Sezary syndrome and melanoma (Figure 6A). In contrast, FTO was negatively associated with pathways involved in adipogenesis, neuroglial cell activity, and immune response (Figure 6B). Similarly, YTHDC1 was positively associated with pathways related to tumorigenesis and cancer progression, including Sezary syndrome, anaplastic thyroid carcinoma, and breast cancer, as well as pathways involved in neurotransmission (Figure 6C). Conversely, YTHDC1 was negatively associated with pathways related to adipogenesis, cell quiescence, and hematological malignancies such as acute myeloid leukemia (Figure 6D). These findings suggest that key m6A regulators are associated with multiple biological processes, including RNA metabolism, immune regulation, and cellular signaling pathways.

### 3.6 Construction of the miRNA-gene-TF interaction network

**Figure 7:**
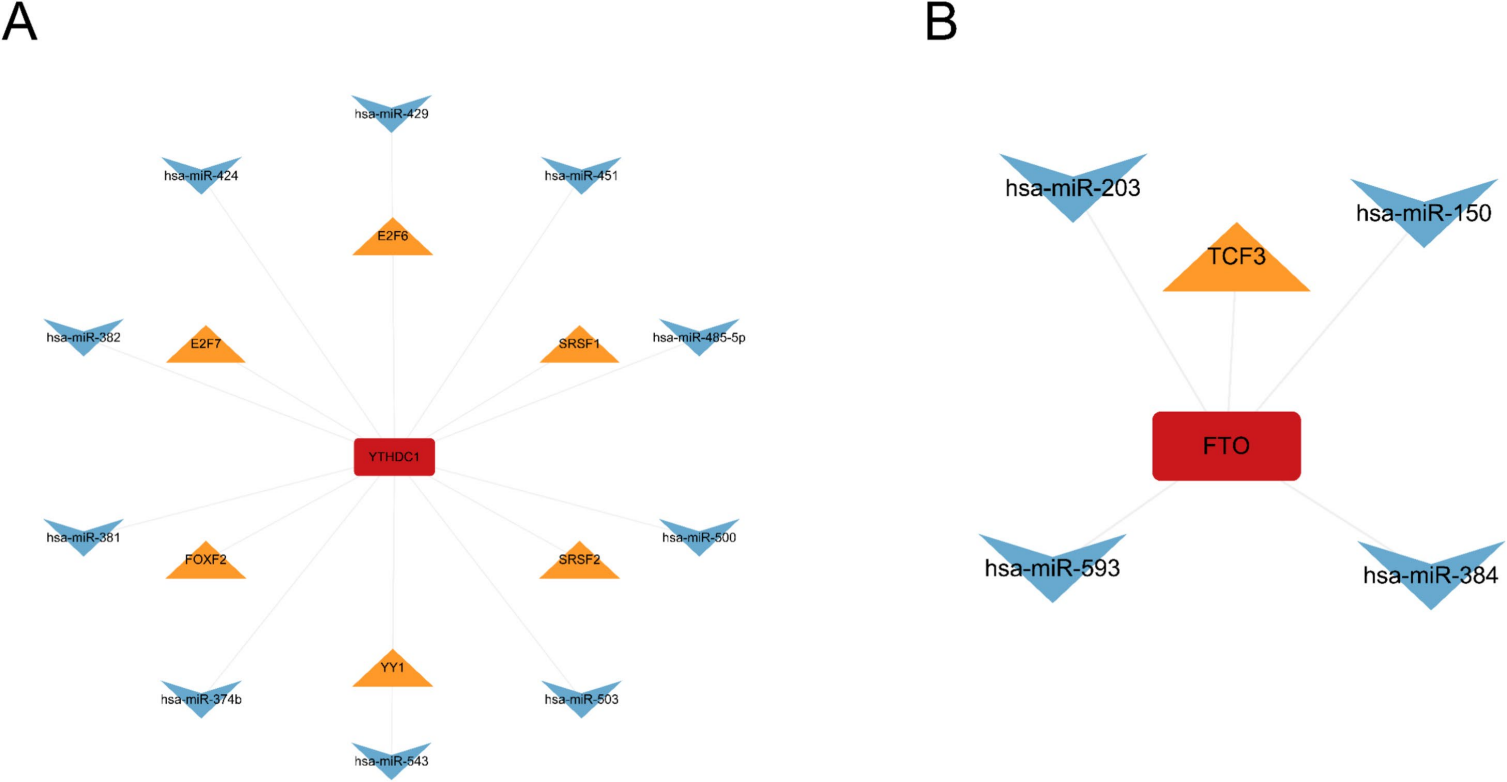
miRNA-mRNA-TF interaction networks of key m^6^A regulators. **(A, B)** Interaction networks of YTHDC1 **(A)** and FTO **(B)**. miRNAs represented by circles, TFs by triangles, and hub genes by rectangles.

To investigate the regulatory relationships among key m6A regulators, miRNAs, and TFs, a miRNA–gene–TF interaction network was constructed for FTO and YTHDC1. The initial network analysis indicated that YTHDC1 was potentially regulated by 22 TFs, 46 miRNAs, and 6 proteins, while FTO was regulated by 1 TF and 4 miRNAs (Figure 7B). To improve the clarity and interpretability of the network, a reduced and more concise subnetwork was constructed by selecting a subset of TFs and miRNAs for visualization purposes. As shown in Figure 7A, the simplified network highlights representative regulatory interactions centered on YTHDC1, illustrating the interplay among TFs, miRNAs, and the hub regulator. The complete interaction network for YTHDC1 is provided in the Supplementary Figure S1.

### 3.7 Single-cell RNA-seq analysis

**Figure 8:**
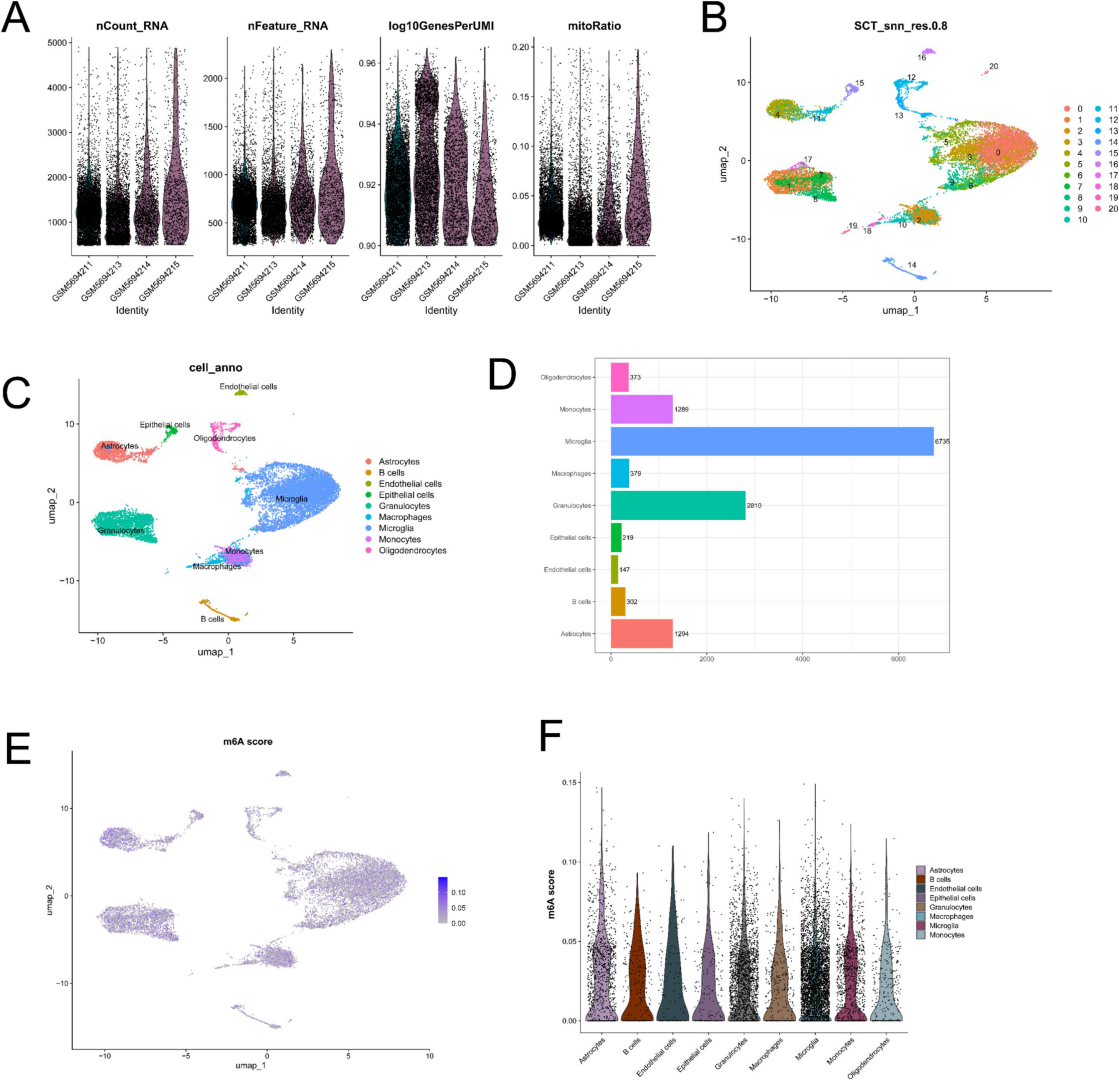
Single-cell RNA seq analysis of the GSE189070 dataset. **(A)** Violin plots of quality control metrics (nCount_RNA, nFeature_RNA, log10GenesPerUMI, and mitoRatio). **(B)** UMAP plot showing cell clustering based on the SCT method (resolution = 0.8). **(C)** UMAP plot of cell types annotation based on canonical marker genes. **(D)** Bar plot of the proportion of each cell type. **(E)** UMAP visualization of m6A scores across individual cells, calculated using the “AUCell” method based on a predefined gene set of m6A regulators. **(F)** Violin plot showing the distribution of m6A scores across different cell types.

Single-cell RNA sequencing data were processed and analyzed using the “Seurat” package. Low-quality cells were filtered based on the following criteria: nFeature_RNA > 200, percent.mt < 25%, nCount_RNA > 200, and nCount_RNA < 5,000, resulting in a total of 14,297 cells (Figure 8A). Dimensionality reduction using UMAP identified 21 distinct cell clusters (Figure 8B). These cluster were annotated into major cell types, including astrocytes, B cells, endothelial cells, epithelial cells, granulocytes, macrophages, microglia, monocytes, and oligodendrocytes (Figure 8C). The proportion of each cell type is shown in Figure 8D. To evaluate m6A regulatory activity at the single-cell level, “AUCell” was applied based on the expression of 23 m6A regulators. The UMAP plot illustrates the distribution of m6A scores across individual cells (Figure 8E). Among the identified cell types, microglia and astrocytes exhibited higher m6A activity, whereas B cells showed relatively lower activity (Figure 8F).

### 3.8 Expression patterns of *Fto* and *Ythdc1* across cell types

**Figure 9:**
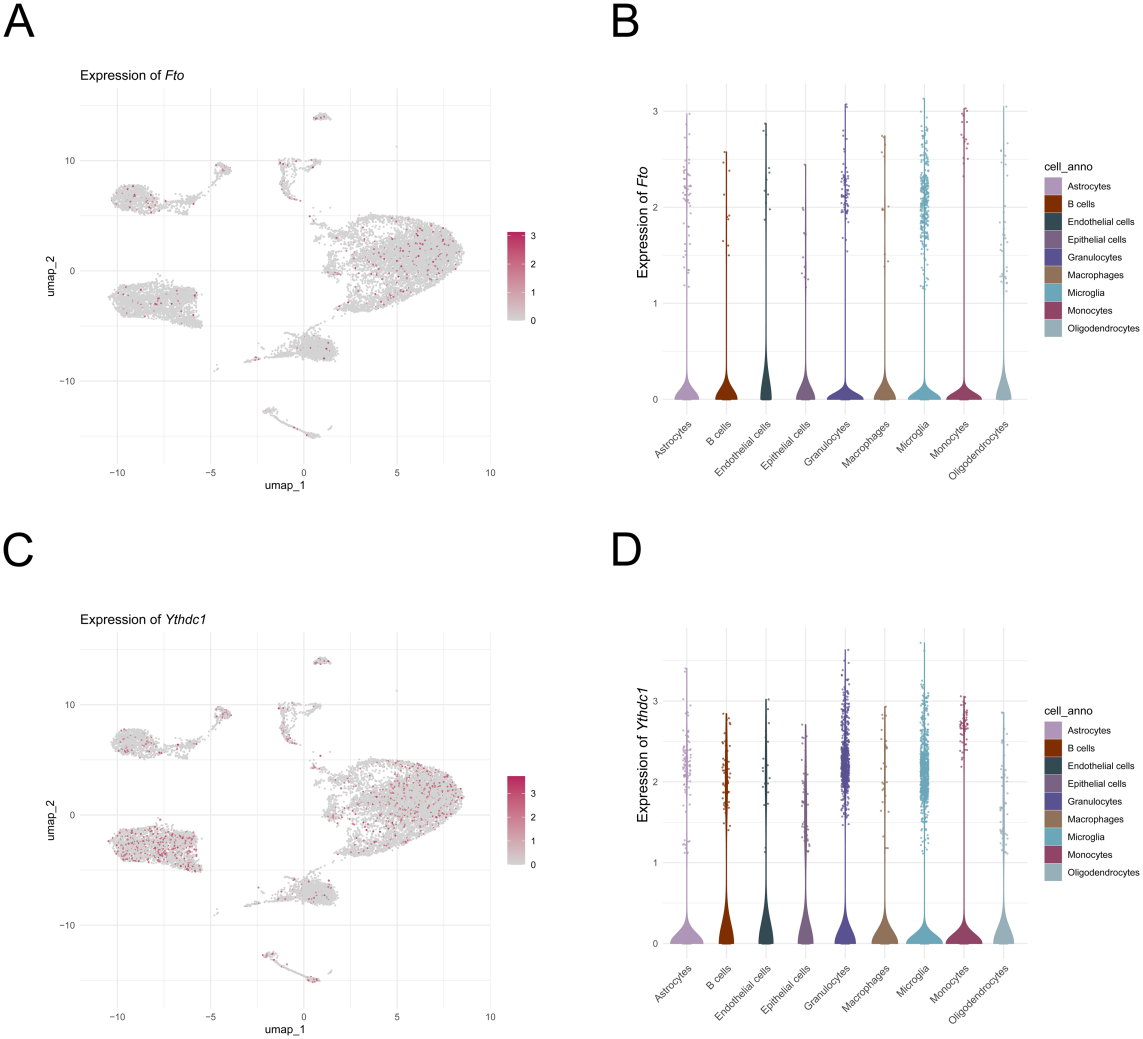
Expression patterns of key m^6^A regulators across cell types. **(A, C)** UMAP plot showing the expression of *Fto* **(A)** and *Ythdc1* **(C). (B, D)** Violin plot showing the expression levels of *Fto* **(B)** and *Ythdc1* **(D)** in different cell types.

To characterize the cell type–specific expression patterns of m6A regulators in the spinal cord, the expression of *Fto* and *Ythdc1* was analyzed at single-cell resolution. UMAP projections showed that both *Fto* (Figure 8A) and *Ythdc1* (Figure 8C) were broadly expressed across multiple cell clusters, with distinct enrichment patterns among different cell types. Violin plot further illustrated the expression levels of *Fto* and *Ythdc1* across cell populations (Figures 8B, D). *Fto* exhibited relatively higher expression in astrocytes, microglia, and granulocytes, whereas lower expression was observed in B cells and epithelial cells (Figure 8B). Similarly, *Ythdc1* was widely expressed across all analyzed cell types, with relatively higher expression in astrocytes, microglia, and granulocytes, and lower expression in epithelial cells (Figures 8D, 9).

### 3.9 *In vivo* validation of key m6A regulators

**Figure 10:**
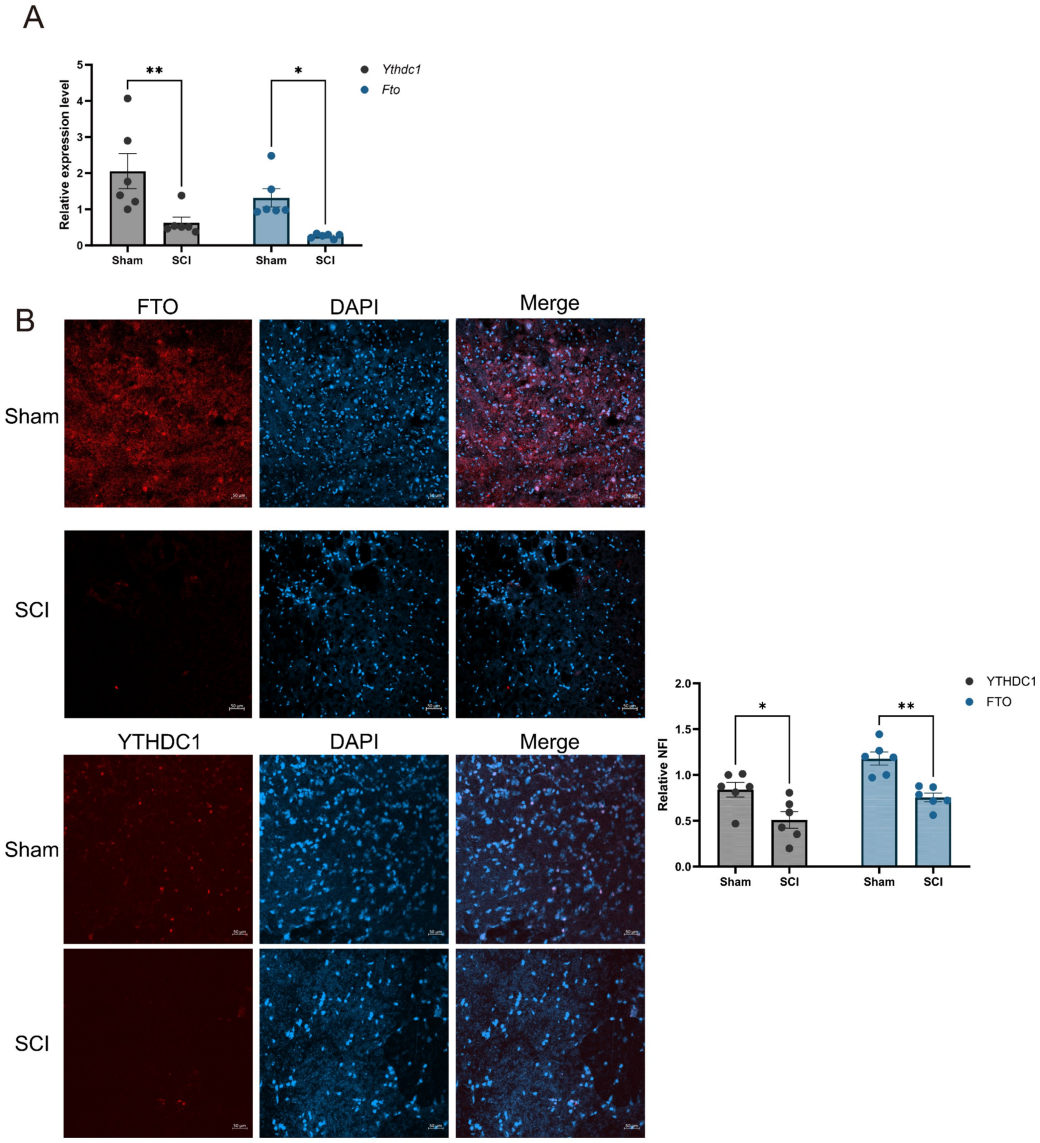
Validation of key m^6^A regulators in Sham and SCI groups. **(A)** qRT-PCR analysis of *F*TO and *Y*THDC*1* expression in rat spinal cord tissues. **(B)** Representative immunofluorescence images showing FTO (red) and YTHDC1 (red) expression in spinal cord tissues from Sham and SCI groups. Scale bar = 50 μm. *p < 0.05, **p < 0.01.

The expression of FTO and YTHDC1 in rat spinal cord tissues was evaluated using qRT-PCR and IF staining. qRT-PCR analysis showed that the mRNA levels of *Fto* and *Ythdc1* were significantly decreased in the SCI group compared with the Sham group (Figure 10A). Consistently with these findings, IF staining demonstrated reduced expression of FTO and YTHDC1 in spinal cord tissues following SCI (Figure 10B). These results are consistent with the bioinformatics analysis, indicating that FTO and YTHDC1 are downregulated in SCI.

## 4 Discussion

SCI represents a major health challenge, characterized by severe neurological deficits that significantly impair quality of life and impose substantial economic burdens on healthcare systems (29). The pathophysiology of SCI is highly complex, involving multiple processes such as inflammation, neuronal apoptosis, and impaired axonal regeneration, which collectively contribute to disease progression and severity (30). Despite advances in therapeutic strategies, including surgical interventions and neuroprotective agents, functional recovery remains limited (31–33). Therefore, further studies are urgently needed to elucidate the underlying molecular mechanisms and identify novel therapeutic targets to improve outcomes for patients with SCI.

Accumulating evidence highlights the critical role of m6A modification in the pathogenesis of various neurological disorders, including neurodegenerative diseases (34–36). Furthermore, previous studies have demonstrated that the disruption of m6A modification accelerates the progression of SCI (14, 37, 38). In this context, the present study aimed to investigates the role of m6A RNA regulators in SCI.

Differential expression analysis revealed significant alterations in 14 m6A regulators between SCI and healthy controls, suggesting dysregulation of m6A methylation in SCI. In addition, the m6A score was markedly increased in SCI samples, further supporting the involvement of m6A modification in SCI pathogenesis. Among these regulators, IGFBP2 was upregulated, whereas WTAP, RBM15B, ZC3H13, METTL16, FTO, YTHDC1, YTHDC2, YTHDF2, FMR1, LRPPRC, HNRNPA2B1, IGFBP3, and RBMX were downregulated, indicating coordinated regulatory alterations.

Functional enrichment analysis showed that these regulators are mainly involved in mRNA metabolic processes, mRNA splicing, and RNA processing. These findings suggest that m6A regulators may influence post-transcriptional regulation in SCI. m6A, as the most abundant RNA modification in eukaryotes, plays a critical role in RNA stability, translation, splicing, and localization (39, 40), and has been implicated in various neurological disorders and central nervous system injuries (41). Following SCI, secondary injury processes such as neuroinflammation, neuronal apoptosis, and oxidative stress contribute to tissue damage and functional impairment. The enrichment of RNA metabolism and splicing-related pathways indicates that m6A regulators may modulate gene expression involved in inflammatory responses and neuronal survival. Consistently, previous studies have shown that dysregulation of RNA processing affects neuronal regeneration and axonal repair after SCI (42).

KEGG analysis further revealed enrichment in pathways such as p53 signaling pathway and cellular senescence. Activation of p53 signaling is known to induce cell cycle arrest and apoptosis under stress conditions and has been implicated in neuronal apoptosis following SCI (43). In addition, cellular senescence has been recognized as a contributor to chronic inflammation and tissue degeneration after SCI (44). These findings suggest that m6A regulators may participate in SCI progression by regulating stress responses and inflammatory signaling pathways. Collectively, these results indicate that m6A regulators may contribute to the development and progression of SCI through modulation of RNA metabolism and key signaling pathways involved in neuronal injury and repair. Using machine learning algorithms, 14 differentially expressed m6A genes were narrowed down to 8 candidate regulators, among which only FTO and YTHDC1 were further validated in the GSE45006 dataset.

Fat mass and obesity-associated protein (FTO) is a key demethylase that regulates the dynamic balance of m6A modification (45). Previous studies have demonstrated that FTO exerts neuroprotective effects in various neurological conditions. For example, FTO promotes neuroprotection after stroke by demethylating c-Jun (46), while its deficiency exacerbates age-related depression-like behaviors and cognitive impairment (47). In addition, FTO-mediated m6A demethylation of OTUB1 stabilizes SLC7A11, thereby reducing ferroptosis in cerebral ischemia/reperfusion injury (48). YTH N6-Methyladenosine RNA Binding Protein C1 (YTHDC1), a nuclear m6A reader, is involved in multiple aspects of RNA metabolism. It has been reported to regulate mRNA splicing, nuclear export, and transcription during muscle stem cell activation (49), and to protect against RNA damage-induced DNA breaks through interaction with the THO complex (50). Moreover, YTHDC1 has been shown to alleviate oxidative stress injury and exert protective effects in tissue injury models (51, 52). Functionally, silencing YTHDC1 increases apoptosis and impairs tissue repair capacity (53). In the present study, both FTO and YTHDC1 was significantly downregulated in SCI rats compared with the Sham group, suggesting that reduced m6A regulatory activity may contribute to SCI pathogenesis.

To further explore the potential biological roles of FTO and YTHDC1 in SCI, single-gene GSEA was performed. The results indicated that these m6A regulators are associated with multiple biological processes, including immune responses, neuroglial activity, and metabolic regulation. These pathways are closely related to the secondary injury cascade following SCI, which involves complex interactions among inflammation, glial activation, and neuronal damage. The enrichment of immune- and neuroglial-related pathways suggests that dysregulation of m6A regulators may influence inflammatory signaling and glial responses after injury. Consistently, previous studies have shown that excessive inflammation and glial activation play critical roles in secondary spinal cord damage (54). Mechanistically, FTO functions as an m6A demethylase that regulates RNA stability and translation efficiency by removing m6A modifications from target transcripts (55). Dysregulation of FTO may therefore affect the expression of genes involved in inflammatory signaling and neuronal survival. In contrast, YTHDC1 acts as a nuclear m6A reader that regulates RNA splicing and nuclear export of methylated transcripts (56). Altered YTHDC1 expression may consequently influence the processing and localization of m6A-modified mRNAs involved in immune and neurobiological pathways. However, it should be noted that ssGSEA estimates the relative enrichment of immune cell populations based on gene signatures and does not directly reflect absolute immune cell abundance.

Immune infiltration analysis revealed increased levels of neutrophils and macrophages, along with decreased levels of NK cells and T cells in SCI samples. The elevated presence of neutrophils and macrophages indicates an enhanced innate inflammatory response following SCI, as these cells release pro-inflammatory cytokines and contribute to secondary injury and neuronal damage. Macrophages exhibit functional heterogeneity, with pro-inflammatory M1 macrophages promoting tissue damage, whereas M2 macrophages are associated with tissue repair and regeneration. In contrast, the reduced infiltration of NK and T cells may reflect impaired adaptive immune responses and immune dysregulation after SCI, which can affect immune surveillance and tissue repair processes (57, 58). Correlation analysis showed that FTO expression was positively associated with adaptive immune cell populations and negatively associated with pro-inflammatory cells such as neutrophils and activated dendritic cells, while YTHDC1 exhibited a similar pattern. These results suggest that m6A methylation may be involved in modulating immune responses following SCI. Furthermore, the associations between FTO and YTHDC1 expression and immune cell populations indicate that m6A regulators may participate in regulating immune cell dynamics and could serve as potential biomarkers for neuroinflammation severity in SCI.

Single-cell RNA sequencing analysis revealed cell-type-specific expression patterns of m6A regulators across spinal cord cell populations. Elevated m6A activity was observed in microglia and astrocytes, while B cells exhibited relatively lower activity, indicating heterogeneous epitranscriptomic regulation in SCI. Consistently, *Fto* and *Ythdc1* showed relatively higher expression in astrocytes, microglia, and granulocytes. Microglia, the resident immune cells of the central nervous system (CNS), play a crucial role in mediating secondary injury following SCI (59). Upon activation, microglia rapidly release pro-inflammatory cytokines, such as IL-1β, TNF-*α*, and IL-6, which initiating local inflammatory response (60, 61). Astrocytes are key contributors to glial scar formation after SCI. In response to injury signals, they proliferate and migrate to the lesion site, where they interact with microglia and other cell types to form glial and fibrotic scars (62). In peri-lesional regions, astrocytes also remodel the extracellular matrix by producing inhibitory molecules such as chondroitin sulfate proteoglycans (CSPGs) and matrix proteins, including collagen and fibronectin. While these processes help limit inflammation, they simultaneously inhibit axonal regeneratio (63, 64). Oligodendrocytes, which are responsible for myelination in the CNS, contribute to axonal repair after SCI. Injury induces the expansion of NG2^+^ oligodendrocyte precursor cells, which migrate to the lesion site and differentiate into myelinating oligodendrocytes, thereby promoting remyelination (65). Collectively, these findings highlight the complex and cell type–specific roles of m6A regulators in SCI and suggest that epitranscriptomic regulation may contribute to the diverse cellular responses following injury.

Regulatory networks analysis revealed the upstream regulatory landscape of m6A regulators, showing that YTHDC1 is potentially regulated by 22 transcription factors and 46 microRNAs, whereas FTO is regulated by one transcription factor and four microRNAs. These findings suggest a more complex regulatory network for YTHDC1 compared with FTO, indicating differential regulatory mechanisms among m6A regulators. Further investigation of key miRNAs and transcription factors may help to clarify the regulatory interactions governing m6A modification and their roles in SCI pathogenesis. In addition, potential feedback interactions between transcription factors and m6A regulators may contribute to the dynamic regulation of gene expression during SCI progression.

Experimental validation using qRT-PCR and IF analyses confirmed the downregulation of FTO and YTHDC1 in the SCI rat model. However, behavioral assessments, such as the Basso, Beattie, and Bresnahan (BBB) locomotor rating scale, which are commonly used to evaluate functional recovery after SCI, were not included in the present study. Future studies incorporating functional behavioral assessments may provide additional insight into injury severity and recovery outcomes.

This study integrates bioinformatics analysis with experimental validation, supporting the reliability of machine learning–based predictions for identifying key m6A regulators in SCI. The exclusion of IGFBP2 due to inconsistent expression across datasets highlights the importance of cross-validation and the challenges associated with candidate gene selection.

Our findings suggest that FTO and YTHDC1 are involved in immune regulation and pathway dysregulation in SCI, indicating a potential role of m6A modification in disease progression. Future studies are needed to validate these findings in human tissues and to further elucidate the underlying molecular mechanisms. These efforts may contribute to the development of m6A-targeted therapeutic strategies for SCI and related neurological disorders.

SCI is characterized by dynamic molecular and cellular changes across distinct pathological phases. In this study, bioinformatic analyses integrated samples from multiple post-injury timepoints to identify common transcriptional features associated with SCI. *In vivo* validation was performed at 3 days post-injury, corresponding to the early secondary injury phase marked by pronounced inflammation and active molecular remodeling. However, the expression and functional roles of m6A regulators, such as FTO and YTHDC1, may vary across different stages of injury progression. Future studies incorporating additional timepoints are required to better characterize the temporal dynamics of m6A-mediated regulation in SCI. These findings also suggest that therapeutic strategies targeting m6A regulators should consider the timing of intervention, as modulation during early inflammatory phases may have different effects compared with later stages of tissue repair.

From a therapeutic perspective, m6A regulators represent promising but context-dependent targets. The role of FTO appears to vary across disease settings. While FTO has been reported to function as an oncogenic factor in multiple cancers and can be targeted by small-molecule inhibitors (66, 67), our findings indicate reduced FTO expression in SCI, suggesting a potential protective role in inflammatory conditions. Therefore, therapeutic strategies targeting FTO may require careful context-specific design, and restoring its activity rather than inhibiting it could be beneficial in SCI. Notably, although FTO inhibitors have been extensively studied in cancer models, their effects in SCI remain unclear and require further investigation. In contrast, targeting m6A readers such as YTHDC1 remains challenging due to the lack of specific pharmacological agents. Potential approaches include modulating RNA–protein interactions, developing small molecules targeting reader domains, or regulating upstream signaling pathways. These strategies require further validation in SCI models to determine their therapeutic potential. Collectively, these findings highlight the complexity of m6A-based therapeutic strategies and emphasize the importance of disease-specific and context-dependent targeting.

Several limitations of this study should be acknowledged. First, although FTO and YTHDC1 were identified as dysregulated m6A regulators in SCI, the current findings are primarily based on transcriptomic analyses and expression validation, lacking direct functional evidence. Further studies, including gain- and loss-of-function experiments, are needed to clarify their causal roles in SCI pathogenesis. Second, the sample size in animal experiments was relatively small (n = 6 per group), which may limit the generalizability of the findings, despite *post hoc* power analysis suggesting adequate statistical power. Third, the absence of longitudinal clinical samples limits the ability to fully characterize temporal dynamics following SCI. Fourth, although multiple GEO datasets were integrated, potential batch effects between datasets (GSE151371 and GSE45006) may introduce biases. Finally, the lack of m6A-seq validation limits direct confirmation of methylation changes and their functional relevance.

## 5 Conclusion

In conclusion, this study identifies FTO and YTHDC1 as key m6A regulators associated with spinal cord injury and highlights their potential roles in immune dysregulation. Through integrative analysis, we provide insights into the molecular mechanisms underlying SCI. The identification of microglia and astrocytes as major cell types associated with m6A activity further suggests cell type–specific regulatory roles in SCI. Future studies are needed to validate these findings in human tissues and to further elucidate the molecular mechanisms of m6A regulation in spinal cord injury.

## Data Availability

The original contributions presented in the study are included in the article/Supplementary material, further inquiries can be directed to the corresponding author.

## Footnotes

1
https://www.networkanalyst.ca
