# Supplementary figures and images for: Comprehensive analysis of m6A RNA methylation regulators and the immune microenvironment in spinal cord injury
Source: Front Neurol. 2026 May 20;17:1759661. doi: 10.3389/fneur.2026.1759661 (PMC13230131; doi:10.3389/fneur.2026.1759661)

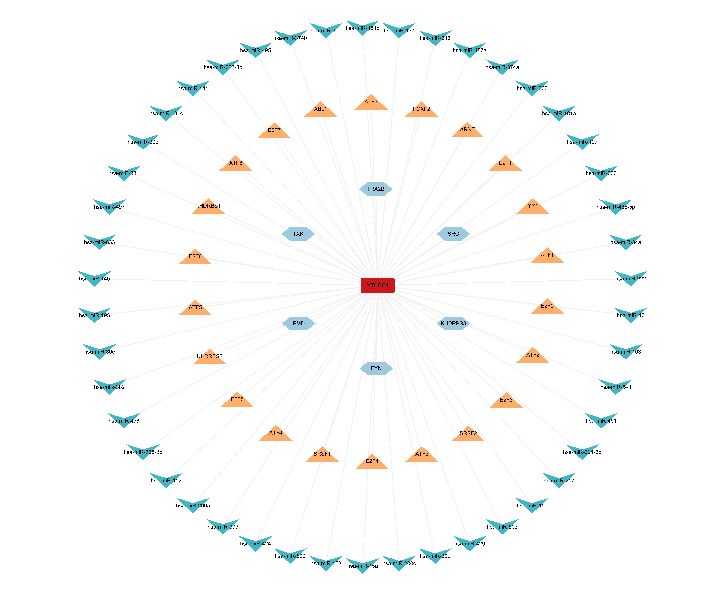


Supplementary Figure S1. Full miRNA–gene–TF interaction network of YTHDC1.

Supplement: Supplementary file 1 [file Supplementary_file_1.DOCX]
